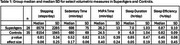# Exploring variability in daily health‐related behaviors as a feature of exceptional cognitive aging in people 80+ years of age: Early findings from the SuperAging Research Initiative

**DOI:** 10.1002/alz70861_108450

**Published:** 2025-12-23

**Authors:** Karen Van Ooteghem, Kit B. Beyer, Ivan Culum, Benjamin F Cornish, Andrew Lim, Richard H. Swartz, Desmond O. Oklikah, Emily Narayan, Elizabeth Finger, Amanda Cook Maher, Felicia C. Goldstein, Adam Martersteck, Ozioma C. Okonkwo, Rhiana Schafer, Emily J Rogalski, William E. McIlroy, Angela C. Roberts, The SuperAging Research Initiative

**Affiliations:** ^1^ University of Waterloo, Waterloo, ON Canada; ^2^ Canadian Centre for Activity and Aging, London, ON Canada; ^3^ Western University, London, ON Canada; ^4^ McMaster University, Hamilton, ON Canada; ^5^ Sunnybrook Research Institute, Toronto, ON Canada; ^6^ Temerty Faculty of Medicine, University of Toronto, Toronto, ON Canada; ^7^ University of Toronto, Toronto, ON Canada; ^8^ Lawson Health Research Institutes, London, ON Canada; ^9^ University of Western Ontario, London, ON Canada; ^10^ University of Michigan, Ann Arbor, MI USA; ^11^ Emory University School of Medicine, Atlanta, GA USA; ^12^ Healthy Aging & Alzheimer’s Research Care (HAARC) Center, University of Chicago, Chicago, IL USA; ^13^ University of Wisconsin‐Madison School of Medicine and Public Health, Madison, WI USA

## Abstract

**Background:**

The SuperAging Research Initiative employs wearable technologies to evaluate whether ‘SuperAgers’ (individuals aged 80+ with episodic memory comparable to those 2‐3 decades younger) exhibit preserved biological and physiological complexity compared to Controls. This preliminary analysis examines variability in early group‐level data and considers alternate methods for capturing variability and complexity in daily health‐related activities.

**Method:**

Data collection and analysis are ongoing. Participants wear wrist and ankle inertial measurement units and a trunk sensor (accelerometer with electrocardiography) 24h daily over 14 days, with scheduled wear breaks. To date, 127 participants have completed device wearing. Volumetric measures of physical activity, mobility, and sleep are derived from limb devices using a custom analytics pipeline (NiMBalWear) validated for use with older adults (Beyer et al. 2024). In this analysis, group level data from 61 participants (42% SuperAgers, 60% female, mean age 85±4.8 years) were compared using independent t‐tests (α=0.05).

**Result:**

Overall, device wear compliance has been high (>90%) and data loss limited. Across all 61 participants, median (SD) sedentary time was 684 (93) min/day, moderate‐to‐vigorous physical activity (MVPA) time was 24.5 (29.0) min/day, step count was 8,232 (4,021) steps/day, sleep duration was 6.94 (1.05) hr/day, and sleep efficiency was 0.83 (0.09). There were no significant differences in group median or median SD between SuperAger and Control participants (Table 1). Notably, there was a high degree of variability in both groups, consistent with our study of young‐older adults with and without neurodegeneration (under review). Day‐to‐day variability often exceeded between‐participant variability. Range of within‐participant variability (SD) was 19‐170 min/day for sedentary time, 1‐58 min/day for MVPA time, 673‐5,860 steps/day for step count, 0.3–1.9 hr/day for sleep duration, and 2‐16% for sleep efficiency.

**Conclusion:**

Objective capture of health‐related behaviours in 80+ year olds has been rare but is important, highlighted by the variability observed among study participants. While results should be interpreted cautiously, volumetric measures of behavior do not appear to differentiate SuperAgers from Controls. This underscores the necessity for further investigation into the structure and relationship of behavioural patterns using more advanced measures of variability (e.g. ApEN, LZC, Gini index), which are underway for this unique dataset.